# Adverse Cardiovascular Effects with Acute Particulate Matter and Ozone Exposures: Interstrain Variation in Mice

**DOI:** 10.1289/ehp.10689

**Published:** 2008-04-22

**Authors:** Ali K. Hamade, Richard Rabold, Clarke G. Tankersley

**Affiliations:** Department of Environmental Health Sciences, Bloomberg School of Public Health, Johns Hopkins University, Baltimore, Maryland, USA

**Keywords:** air pollution, genetic susceptibility, heart rate variability, Toll-like receptor 4

## Abstract

**Objectives:**

Increased ambient particulate matter (PM) levels are associated with cardiovascular morbidity and mortality, as shown by numerous epidemiology studies. Few studies have investigated the role of copollutants, such as ozone, in this association. Furthermore, the mechanisms by which PM affects cardiac function remain uncertain. We hypothesized that PM and O_3_ induce adverse cardiovascular effects in mice and that these effects are strain dependent.

**Study design:**

After implanting radiotelemeters to measure heart rate (HR) and HR variability (HRV) parameters, we exposed C57Bl/6J (B6), C3H/HeJ (HeJ), and C3H/HeOuJ (OuJ) inbred mouse strains to three different daily exposures of filtered air (FA), carbon black particles (CB), or O_3_ and CB sequentially [O_3_CB; for CB, 536 ± 24 μg/m^3^; for O_3_, 584 ± 35 ppb (mean ± SE)].

**Results:**

We observed significant changes in HR and HRV in all strains due to O_3_CB exposure, but not due to sequential FA and CB exposure (FACB). The data suggest that primarily acute HR and HRV effects occur during O_3_CB exposure, especially in HeJ and OuJ mice. For example, HeJ and OuJ mice demonstrated dramatic increases in HRV parameters associated with marked brady-cardia during O_3_CB exposure. In contrast, depressed HR responses occurred in B6 mice without detectable changes in HRV parameters.

**Conclusions:**

These findings demonstrate that important interstrain differences exist with respect to PM- and O_3_-induced cardiac effects. This interstrain variation suggests that genetic factors may modulate HR regulation in response to and recuperation from acute copollutant exposures.

Numerous epidemiology studies have documented associations between particulate matter (PM) and cardiovascular morbidity and mortality (Bell et al. 2005; Dominici et al. 2005). Although the exact mechanisms by which air pollutants may mediate adverse health effects remain unclear, varying the physicochemical properties of PM and the exposure conditions result in different adverse effects on cardiac function, including the neural regulation of the heart (Schulz et al. 2005). In addition, the existence of copollutants and their adverse cardiovascular health effects have not been suitably investigated. For example, a recent human study suggested that PM and ozone influenced cardiac autonomic control (Chuang et al. 2007). In that study, the negative impact of O_3_ on heart rate variability (HRV) was equivalent to or more profound than the effects of PM. The mechanisms for these HRV effects of PM in combination with O_3_ merit further investigation, particularly in animal models.

Although numerous animal studies have examined the cardiac effects of PM, few did so in conjunction with copollutants such as O_3_. In one such study, Watkinson et al. (2001) showed a decreased heart rate [HR; beats per minute (bpm)] due to O_3_ as a single pollutant. Likewise, our laboratory has recently described the strain differences in PM-mediated autonomic neural control of HR between C3H/HeJ (HeJ) and C57Bl/6J (B6) mice (Tankersley et al. 2007). That study suggested that genetic susceptibility factors may be important in the neural regulation of PM-induced HR effects. In the present study, we tested the hypothesis that genetic susceptibility factors play a major role in PM-induced cardiac effects, particularly in the face of O_3_ preexposure. We based this hypothesis on previous findings showing greater pulmonary infiltration of neutrophils in B6 than in HeJ mice after O_3_ exposure by inhalation (Tankersley and Kleeberger 1994). Other studies using these same strains showed increased macrophage activity in B6 mice after inhalation of sulfate-coated CB particles (Ohtsuka et al. 2000). These effects are believed to be mediated in part via the Toll-like receptor 4 (*Tlr4*) signaling pathway. In HeJ mice, the *Tlr4* gene is mutated and not functional (Kleeberger et al. 2000; Poltorak et al. 1998). Alternatively, C3H/HeOuJ (OuJ) mice are coisogenic relative to HeJ mice and demonstrate a fully functional *Tlr4* gene construct and downstream signaling pathway (Qureshi et al. 1999).

The purpose of this study was 3-fold. First, we determined whether there are interstrain differences in HR and HRV characteristics among the B6, HeJ, and OuJ mice. Second, we examined the HR and HRV responses to PM [i.e., carbon black (CB)] after preexposure to filtered air (FA) and O_3_ in HeJ and OuJ mice compared with B6 mice. Third, we more specifically tested the hypothesis that the *Tlr4* gene mutation and signaling pathway modulates the cardiac response to the O_3_CB exposure. This hypothesis is supported by observations showing the activation of TLR4 signaling associated with O_3_-induced lung injury and inflammation (Kleeberger et al. 2000). We found robust interstrain variation in response to PM with preexposure to O_3_, which significantly decreased HR and increased HRV compared with FA. Furthermore, we detected no differences in HR regulation between HeJ and OuJ mice.

## Materials and Methods

### Animals

We purchased male mice of three inbred strains, B6 (*n* = 8), HeJ (*n* = 5), and OuJ (*n* = 6), from Jackson Labs (Bar Harbor, ME). Mice were housed under a 12/12-hr light/dark cycle in an animal facility at the Johns Hopkins Bloomberg School of Public Health. Room temperature was maintained at 21 ± 1ºC (mean ± SE), and regular lab chow and drinking water were provided *ad libitum*. We treated animals humanely and with regard for alleviation of suffering. All experiments were conducted with approval from the Animal Care and Use Committee of the Johns Hopkins University Medical Institutions.

### Surgical procedure

The surgical procedure for transmitter implant (model TA10ETAF20) and the radiotelemetry system (both from Data Sciences International, St. Paul, MN) used to measure core temperature (T_CO_) and HR and to sample electrocardiograph (ECG) recordings have been described elsewhere (Tankersley et al. 2003). Briefly, we anesthetized each animal intraperitoneally with a mixture of 10 mg/mL acepromazine and 100 mg/mL ketamine (1:10) at a dose of 2 μL/g body weight. We removed abdominal and chest fur, applied Betadine to the exposed region of skin, and established a sterile field surrounding the animal. The transmitter was inserted through a midline abdominal incision and sutured to the abdominal muscle; the negative ECG lead was guided through the muscle and directed subcutaneously to the right shoulder. The positive ECG lead, also guided through the muscle, was directed laterally and positioned approximately 1 cm below the rib cage, and both leads were sutured to the muscle tissue in a lead II position in traditional human ECGs. Surgery required 30 min, and recovery from anesthesia occurred within 60–90 min. We allowed mice to recover from surgery for at least 2 weeks before we started data collection, which began at 18–20 weeks of age.

### CB and O_3_ exposure protocol

**Figure 1:**
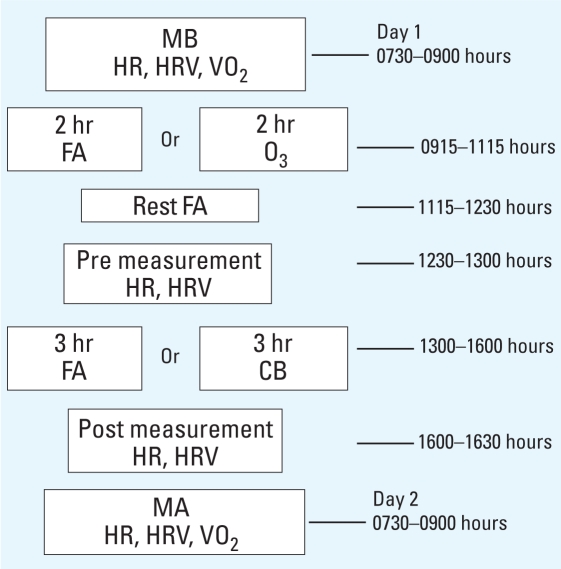
Flow diagram of the timing of measurements obtained immediately before (pre), during, and immediately after the 3-hr exposure (post). Abbreviations: MA, morning after; MB, morning before; VO_2_, volume of oxygen consumption per body weight over time.

We exposed all mice to three different daily exposure protocols, and acclimated them to exposure chambers 1 day before any given exposure. Each exposure protocol lasted 1 day, and the sequence of exposure for each mouse occurred in the following order (Figure 1): *a*) 2 hr FA followed by another 3 hr FA (FAFA); *b*) 2 hr FA followed by 3 hr CB (FACB); and *c*) 2 hr O_3_ followed by 3 hr CB (O_3_CB). The first 2-hr exposure consistently occurred between 0915 and 1115 hours, and this was followed with a second 3-hr exposure occurring between 1300 and 1600 hours. A 75-min recovery period occurred between the two sequential exposures from 1115 to 1230 hours to allow for animal transfer between different exposure chambers (~ 15 min), and animal reacclimation to the second chamber (~ 60 min). The reacclimation period was sufficient to eliminate confounding effects of animal handling on preexposure HR and HRV data collection, which consistently occurred between 1230 and 1300 hours.

Using a subgroup of eight randomly selected mice from the three test strains, we conducted a fourth exposure protocol consisting of 2 hr of O_3_ followed by 3 hr of FA (O_3_FA), using the same rigorous time schedule as described above. This allowed us to determine whether time-dependent HR and HRV changes during the 3-hr exposure were attributable to the added effects of CB exposure or simply due to the recovery from O_3_ preexposure.

### CB and O_3_ exposure assessment

Preexposure to either O_3_ or FA occurred in individual stainless steel chambers for 2 hr. O_3_ was produced by an O_3_ generator using ultraviolet light (Orec, Phoenix, AZ), which we monitored with a 1003-AH Dasibi monitor (Dasibi Environmental, Glendale, CA). The target O_3_ concentration for the 2-hr exposure was 0.5 ppm.

We conducted the second exposure to CB or FA in individual Plexiglas chambers for 3 hr. The CB (Regal 660; density, 1.95 g/cm^3^; specific surface area, 112 m^2^/g; empirical formula, C_910_H_34_O_10_; composition, 96.90% carbon, 1.42% oxygen, 0.30% hydrogen) was aerosolized by a Wright Dust Feeder (BGI, Inc., Waltham, MA). To assess exposure concentration, we collected particles on 25-mm-diameter glass fiber filters for gravimetric analysis using a Mettler Toledo microbalance (Mettler Toledo, Columbus, OH). We also monitored particles with an Aerodynamic Particle Sizer (model 3320; TSI, Shoreview, MN) for mass median aerodynamic diameter (MMAD) and count median diameter (CMD) assessments.

### T_CO_, HR, and HRV measurements

We derived the HR and HRV measurements obtained during and immediately before and after the second 3-hr exposure from 3-min ECG samples collected every 15 min. Each 3-min ECG sample was analyzed using a peak detect algorithm (Data Sciences International, St. Paul, MN), which was achievable in samples lacking motion or body-position artifacts. We examined the resulting tachograms (peak interval vs. time) individually to detect and correct errors in intervals associated with arrhythmias or missed peak detections. Parameters in the time domain consisted of average HR, standard deviation of normal-to-normal intervals (SDNN) as a measure of total HRV, and the root mean square of successive differences between adjacent R-R intervals (rMSSD) as a measure of beat-to-beat HRV. From the frequency domain, we extracted the HRV ratio of low frequency (LF) to high frequency (HF). The LF range was calculated as the area under each density curve from 0.2 to 1.5 Hz, and the HF range was calculated between 1.5 Hz and the Nyquist frequency (HR frequency divided by 2, which was typically between 4 and 5 Hz). We averaged the four individual hourly measurements to represent the HR and HRV responses for each time point during exposure.

In addition to HR and HRV measurements obtained before, during, and after the second 3-hr exposure, we also collected measurements on the morning before (MB) and on the morning after (MA) each exposure protocol. On consecutive mornings, we consistently obtained the measurements during the period between 0730 and 0900 hours. Individual measurements obtained on consecutive mornings represent the average of five data samples collected during each 90-min period. We compared MA and MB measurements to assess whether prolonged cardiac effects were evident after each exposure. Likewise, we assessed the 24-hr circadian pattern of HR and T_CO_ for each animal on a weekly basis to determine whether there were longer-term residual effects of surgery or cumulative effects of repeated exposure protocols. The procedures to assess circadian pattern in mice have been described elsewhere (Tankersley et al. 2003). We summarized individual circadian pattern characteristics by determining the mean, minimum, and maximum values for HR and T_CO_ from each average 24-hr cycle before and after each exposure protocol.

### Oxygen consumption assessment

We repeatedly measured oxygen consumption (VO_2_) on consecutive mornings (MA and MB) using an indirect open-circuit calorimetric system (Oxymax Deluxe; Columbus Instruments, Columbus, OH) in-line with 200 mL cylindrical Plexiglas chambers. We delivered unhumidified compressed air through the chamber under the control of a calibrated flow meter, and adjusted the flow to maintain a small difference between chamber inflow (21% O_2_ in nitrogen gas) and outflow O_2_ concentrations. We dried the air flow out of the chamber with a column of anhydrous calcium sulfate and sampled it for 30 sec for fractional concentrations of O_2_ using a limited-diffusion oxygen sensor (Columbus Instruments). We captured sensor output with data-acquisition software (Oxymax version 5.3; Columbus Instruments) and recorded it with a computer. We conducted gas-analyzer calibrations before each experiment using standardized gas mixtures (Puritan Bennett, Linthicum Heights, MD). We obtained intermittent reference air measurements to correct for sensor drift; we also normalized the VO_2_ data to standard temperature, pressure, and dry conditions and as a function of body weight.

### Oxygen pulse (VO_2_/HR)

We derived the measurement of O_2_ pulse as the ratio of VO_2_ to HR, as an indicator of cardiac stroke volume (SV) (Bhambhani et al. 1994). The following equations show a logical proportionality between O_2_ pulse and SV: If cardiac output (CO) = SV × HR, and Fick’s principle defines CO = VO_2_/(venous O_2_ – arterial O_2_), then SV ≈VO_2_/HR, provided that the O_2_-carrying capacity of blood (venous O_2_ – arterial O_2_) remains relatively constant.

### Data analysis

We present the results as mean ± SE. For results obtained before, during, and after the second 3-hr exposure to either CB or FA, we used a three-way analysis of variance (ANOVA) to evaluate the effects of strain, time, and exposure. We applied adjustments to the effects of time and exposure to account for repeated measures. We also used a similar three-way ANOVA, including adjustments for repeated measures, to evaluate MA and MB results. For results obtained from 24-hr circadian pattern characteristics, we performed a two-way ANOVA adjusted for repeated measures to assess the effects of strain and exposure. We determined significant mean comparisons using the Duncan’s multiple range test with an α of *p* < 0.01 to adjust for the higher probability of type I error in multiple comparisons. Given the absence of statistically detectable differences between HeJ and OuJ mice, we combined these substrains to represent the C3 strain (*n* = 11 mice) and repeated the analyses. We used a similar analytic approach to determine statistically significant differences between O_3_CB and O_3_FA exposures.

## Results

### Exposure assessment

**Figure 2:**
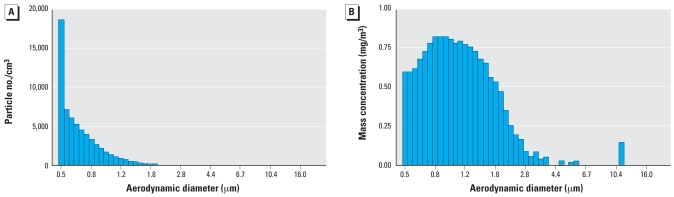
Exposure assessment results shown as the average profile of particle number (*A*) and mass concentration (*B*) for the CB aerosol generated from a Wright Dust Feeder.

Figure 2 shows the average profiles for the number and mass concentrations of the CB exposure aerosol. Generally, animals were exposed to particles that were in the fine mode (PM ≤2.5 μm in aerodynamic diameter). A time-weighted gravimetric analysis of the generated CB aerosol immediately downstream of the exposure chamber showed an exposure concentration of 536 ± 24 μg/m^3^. The particle size distribution of the CB aerosol was characterized by a CMD of 0.7 μm and an MMAD of 1.01 μm, with a geometric standard deviation (GSD) of 1.56 μm. The average O_3_ concentration was 584 ± 35 ppb.

### Circadian pattern characteristics

In addition to measuring body weight, we assessed homeostatic stability after surgery and repeated exposures using weekly measurements of the 24-hr mean, minimum, and maximum for T_CO_ and HR (Table 1). Although a significant strain effect (*F*_df=2_ = 13.1; *p* < 0.01) influenced variation in body weight, no detectable differences among the strains were due to the different exposure conditions. In addition, we found significant variation among the strains in several circadian pattern characteristics, such as the mean daily HR (*F*_df=2_ = 13.0; *p* < 0.01); however, we detected no differences in any circadian pattern characteristics across the three exposure protocols. These findings suggest no longer-term effects of surgery or repeated exposure on the homeostatic stability in these animals. With respect to strain variation, generally T_CO_ was significantly (*p* < 0.01) lower in HeJ mice compared with the other strains. The minimum T_CO_ was significantly (*p* < 0.01) higher in OuJ mice than in the other strains. B6 mice demonstrated a significantly (*p* < 0.01) lower HR compared with the other strains, which was consistent before each exposure protocol.

### MA and MB responses

HR at MA was significantly lower (*F*_df=1_ = 10.2; *p* < 0.01) than HR at MB, independent of strain and exposure effects (Table 2). This effect was particularly apparent in the HR response of B6 mice after O_3_CB exposure. In general, SDNN and rMSSD were significantly (*p* < 0.01) higher in B6 mice than in the other strains, and we detected no effects of exposure associated with these HRV parameters. In contrast, the interaction between exposure and strain in the LF/HF ratio was significant (*F*_df=4_ = 9.8; *p* < 0.01); this was attributable to a significant(*p* < 0.01) decrease in OuJ mice after O_3_CB exposure. Although we observed no significant variation between MA and MB VO_2_ responses, the interactive effect of strain on O_2_ pulse after exposure was significant (*F*_df=2_ = 5.8; *p* < 0.03). Specifically, in B6 mice the MA O_2_ pulse after FACB and O_3_CB exposures was significantly (*p* < 0.01) greater relative to FAFA exposure.

### HR and HRV responses to CB with and without O_3_ preexposure

**Figure 3:**
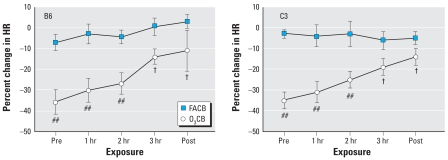
Individual HR responses obtained from 3-min ECG samples in B6 (*n* = 8) and C3 (*n* = 11; 6 OuJ and 5 HeJ) mice. We computed the percent change in HR with respect to FAFA responses for FACB and O_3_CB exposures. Time points represent mean (± SE) percent change in HR immediately before CB exposure (Pre), each hour during exposure (1, 2, and 3 hr), and immediately after CB exposure (Post). ^##^*p* < 0.01 vs. FAFA exposure. ^†^*p* < 0.01 vs. Pre.

The relative changes in HR and HRV responses (i.e., compared with FAFA) during the latter 3-hr period of the FACB and O_3_CB exposures were indistinguishable between HeJ and OuJ mice; hence, we combined these substrains and identify them here as the C3 strain. Figure 3 shows a significant (*F*_df=4_ = 11.4; *p* < 0.01) interaction between the effects of exposure and time on the HR response in B6 and C3 mice. This effect was largely attributable to a significant (*p* < 0.01) decrease in HR responses after the O_3_ preexposure in both B6 and C3 strains. In addition, the recovery in HR during the 3-hr CB exposure was significant (*p* < 0.01) in both strains. However, we detected no strain differences between B6 and C3 mice in either the decline or the recovery of HR associated with O_3_CB exposure. Also, we detected no change in HR during CB exposure after FA preexposure in either strain.

**Figure 4:**
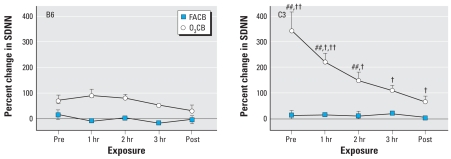
Individual SDNN responses obtained from 3-min ECG samples in B6 (*n* = 8) and C3 (*n* = 11; 6 OuJ and 5 HeJ) mice. We computed the percent change in SDNN with respect to FAFA responses for FACB and O_3_CB exposures. Time points represent mean (± SE) percent change in SDNN immediately before CB exposure (Pre), each hour during exposure (1, 2, and 3 hr), and immediately after CB exposure (Post). ^##^*p* < 0.01 vs. FAFA exposure. ^†^*p* < 0.01 vs. Pre. ^††^*p* < 0.01 vs. B6 strain.

**Figure 5:**
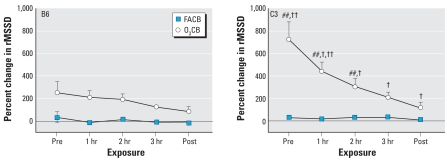
Individual rMSSD responses obtained from 3-min ECG samples in B6 (*n* = 8) and C3 (*n* = 11; 6 OuJ and 5 HeJ) mice. We computed the percent change in rMSSD with respect to FAFA responses for FACB and O_3_CB exposures. Time points represent mean (± SE) percent change in rMSSD immediately before CB exposure (Pre), each hour during exposure (1, 2, and 3 hr), and immediately after CB exposure (Post). ^##^*p* < 0.01 vs. FAFA exposure. ^†^*p* < 0.01 vs. Pre. ^††^*p* < 0.01 vs. B6 strain.

Figures 4 and 5 show the changes in SDNN and rMSSD during CB exposure after either FA or O_3_ preexposure. We observed significant interactive effects of exposure, time, and strain on the SDNN (*F*_df=4_ = 11.5; *p* < 0.01) and rMSSD (*F*_df=4_ = 7.0; *p* < 0.01) parameters. These interactive effects were attributable to significantly (*p* < 0.01) greater changes in SDNN and rMSSD associated with O_3_CB exposure in C3 compared with B6 mice. Although we detected no differences between FACB and O_3_CB exposure protocols in either SDNN or rMSSD for B6 mice, the changes in these HRV parameters were notably (*p* < 0.01) elevated in C3 mice during O_3_CB exposure. In addition, the time course of recovery in these HRV parameters during the 3-hr CB exposure was significant (*p* < 0.01) in only the C3 strain.

**Figure 6:**
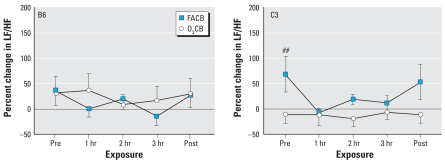
Individual LF/HF ratios obtained from 3-min ECG samples in B6 (*n* = 8) and C3 (*n* = 11; 6 OuJ and 5 HeJ) mice. We computed the percent change with respect to FAFA responses for FACB and O_3_CB exposures. Time points represent mean (± SE) percent change in LF/HF immediately before CB exposure (Pre), each hour during exposure (1, 2, and 3 hr), and immediately after CB exposure (Post). ^##^*p* < 0.01 vs. FAFA exposure.

The interaction of exposure and strain on the changes in LF/HF during CB with either FA or O_3_ preexposure was significant (*F*_df=4_ =7.3; *p* < 0.02; Figure 6). The effect was attributable to a modest difference between FACB and O_3_CB exposure in C3 mice.

### HR and HRV responses after O_3_ with and without CB

**Figure 7:**
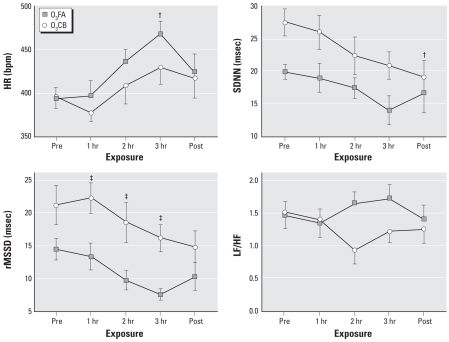
Individual HR responses and HRV parameters obtained from 3-min ECG samples from a random mixture of B6 (*n* = 3) and C3 (*n* = 5) mice after O3 preexposure. Averages at each time point represent the mean (± SE) computed immediately before FA or CB exposure (Pre), each hour during exposure (1, 2, and 3 hr), and immediately after FA or CB exposure (Post). ^‡^*p* < 0.01 vs. O_3_FA exposure. ^†^*p* < 0.01 vs. Pre.

Figure 7 shows HR and HRV responses to either FA or CB after O_3_ preexposure. The significant effect of time on HR (*F*_df=4_ = 5.0; *p* < 0.01) was due, in part, to a significantly more rapid HR recovery associated with O3FA exposure. With respect to SDNN and rMSSD, we observed significant effects of both time (*F*_df=4_ = 5.8 and 5.0, respectively; *p* < 0.01) and exposure (*F*_df=1_ = 11.0 and 9.8, respectively; *p* < 0.02). In particular, rMSSD remained significantly (*p* < 0.01) elevated during the 3-hr exposure to CB relative to FA after preexposure to O_3_.

## Discussion

The results from the present study demonstrate profound strain differences in cardiac function during CB exposure with acute O_3_ preexposure. The most obvious difference between B6 and C3 strains is in the HRV parameters after sequential exposure to O_3_ and CB (Figures 4 and 5). In contrast to a modest effect of O_3_CB in B6 mice, C3 mice showed dramatic changes in SDNN and rMSSD responses, indicating that HR regulation was uniquely different between strains. Despite these strain-specific differences in HRV, relative changes in HR responses were similar between B6 and C3 strains (Figure 3). The strain difference in HRV responses appeared to be dependent on O_3_ preexposure, because FACB exposure did not produce significant changes in these cardiac functional parameters. Further exploratory findings in the same mice exposed to O_3_CB also showed differences in HRV responses compared with O_3_FA exposure (Figure 7). These HRV effects include a notable elevation in rMSSD with O_3_CB accompanied by a delay in HR recovery. These results suggest that a sequential exposure to CB altered HR regulation after O_3_ preexposure. Therefore, the most salient finding in the present study points to B6 and C3 strain differences in HR regulation in mice exposed sequentially to O_3_ and CB. This finding suggests that robust genetic determinants can variably alter HR regulatory mechanisms when adapting to these copollutants.

**Figure 8:**
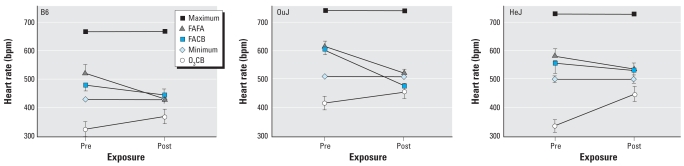
Average strain-specific HR responses for B6 (*n* = 8), OuJ (*n* = 6), and HeJ (*n* = 5) mice computed immediately before FA or CB exposure (Pre) and immediately after FA or CB exposure (Post), derived from the second 3-hr exposure to FAFA, FACB, and O_3_CB exposure protocols. The strain-specific limits (minimum and maximum) in HR during the circadian cycle are also shown as references.

We made several other important observations in the present study. The comparison between OuJ and HeJ mice, for example, did not produce any detectable differences in HR regulation during either FACB or O_3_CB exposure. Therefore, these results suggest that the *Tlr4* mutation in HeJ mice relative to OuJ mice did not affect the HR regulatory adaptive mechanisms associated with O_3_ or CB exposure. Another important observation suggests that HR responses to O_3_CB exposure among the strains were substantially below their strain-specific minimum HR associated with a normal circadian pattern (Figure 8). The strain-specific lower minimum and maximum HR characteristics seen in B6 relative to HeJ mice have been previously described (Tankersley et al. 2002, 2007). Here, the magnitude of the bradycardic response to O_3_CB exposure can be as much as 160 bpm below the normal minimum, as seen in HeJ mice. In contrast, the same bradycardic response to O_3_CB exposure is only 40 and 100 bpm below the normal minimum HR of OuJ and B6 mice, respectively. Although the precise mechanisms underlying this bradycardic response remain unclear, it is obvious from the strains examined in the present study that O_3_ preexposure does not lead to tachycardia; that is, HR responses requiring increased sympathetic tone and/or withdrawal of parasympathetic tone. It is also clear that the bradycardic response to O_3_ preexposure is associated with dramatic increases in SDNN and rMSSD in C3 mice and not in B6 mice, suggesting that the O_3_-induced bradycardia is likely dissociated from altered HRV characteristics. These new insights merit future studies to elucidate the specific cardiac effects of O_3_ exposure alone and in combination with PM. These studies should also consider the importance of genetic susceptibility factors.

In a previous study, our laboratory showed that B6 mice have significant withdrawal of cardiac parasympathetic tone during acute CB exposure (Tankersley et al. 2007). Specifically, CB-induced HR responses were significantly elevated compared with FA responses in B6 mice after sympathetic blockade using pro-pranolol. This elevated HR response was accompanied by a significantly reduced rMSSD, a measure of beat-to-beat HRV. Similar findings were not apparent in C3 mice. The results of the present study show that the O_3_-induced depression in HR was accompanied by a dramatic increase in rMSSD in C3 mice, but not in B6 mice. Although it is difficult to resolve the specific mechanisms leading to O_3_-induced bradycardia, it is reasonable to conclude that increases in parasympathetic tone and/or decreases in sympathetic tone lead to such bradycardia. The strain differences between B6 and C3 mice likely center on the differential balance between these two neural inputs. That is, one strain relies predominantly on withdrawal of sympathetic tone, whereas the HR response of the other strain is principally derived from a greater imposition of parasympathetic tone. Variation in HR regulation involving the balance of parasympathetic and sympathetic inputs may also lead to differences in HR and HRV responses between O_3_CB and O_3_FA exposures.

The changes in HR and HRV responses associated with the sequential effects of O_3_ and CB are generally confined to the time period immediately before and after exposure. Results comparing MA with MB suggest that modest but significant reductions in HR and HRV responses after O_3_CB are present in B6 and OuJ mice, respectively (Table 2). In addition, B6 mice showed a greater O_2_ pulse in the MAFACB exposure. Although it is unclear whether these findings are attributable to prolonged exposure effects, longer-term cardiac compensation may be occurring, especially in B6 mice. An increase in O_2_ pulse, for example, is coincident with a decreased HR, suggesting that CB exposure provokes a greater O_2_ delivery for each stroke of the heart. This elevated response may also imply that SV was elevated in CB-exposed B6 mice. If O_2_ pulse represents a surrogate for relative changes in SV, the results of the present study suggest that CB-induced changes in the heart include longer-term effects of a greater SV unique to B6 mice.

The present study suggests that O_3_-induced effects on HRV are greater than the same responses after CB. Although it mayappear that the O_3_-induced cardiac effects are more potent than those associated with CB exposure, comparable doses may be a limitation. This is especially difficult to ascertain in the absence of clear mechanisms of action underlying the cardiac effects of these copollutants. Evidence in the air pollution epidemiology supports the notion that O_3_ may be as effective as or more potent than PM with respect to risk of mortality and morbidity. For example, Chuang et al. (2007) showed larger reductions in HRV indices associated with PM using a 1-day average, but also demonstrated larger reductions using 2- and 3-day averages for O_3_. Other studies showed changes in HRV that were comparable for increases in O_3_ and PM (Gold et al. 2000; Schwartz et al. 2005a). Likewise, another study (Hong et al. 2002) showed the relative risk (RR) of cardiovascular mortality associated with increases in ambient O_3_ to be almost 2-fold higher (RR =2.9) than that associated with increases in PM ≤10 μm in aerodynamic diameter (RR = 1.5).

Although the mechanisms behind O_3_-induced cardiac changes remain unclear, onev hypothesis proposes that O_3_ leads to increased oxidative stress, resulting in lung inflammatory and permeability changes in addition to airway hyperreactivity (Tankersley and Kleeberger 1994; Zhang et al. 1995). These acute indicators of lung injury may provoke vagally mediated effects on the heart. The B6 strain is known to endure a greater O_3_-induced lung inflammatory and permeability response relative to C3 mice (Tankersley and Kleeberger 1994). However, in the present study, B6 mice showed a modest, statistically insignificant HRV response compared with the dramatic increase in C3 mice. These data suggest that the depression in HR and changes in HRV are not necessarily associated with O_3_-induced lung inflammation, changes in permeability, or airway hyperreactivity. Results in the present study also appear to diminish the possibility that the *Tlr4* signaling pathway is important in cardiac changes induced by O_3_. Likewise, Jimba et al. (1995) showed no differences in O_3_-induced bradycardic effect in rats depleted of pulmonary C-fibers by neonatal capsaicin treatment. This study suggests that C-fiber stimulation of vagal afferents is not a likely mechanism linking O_3_-induced lung injury to altered HR regulation.

One way to test the interactive effects of air-pollutant–induced lung injury on adverse cardiac changes is to evaluate the impact of blocking the production of reactive oxygen species in the present animal model. Anti-oxidant effects have been demonstrated in an epidemiology study showing that omega-3 fatty acid supplementation can eliminate HRV changes associated with PM exposure (Romieu et al. 2005). Future studies might also employ longer-term exposures to CB and O_3_ to evaluate chronic cardiac changes. In addition, studies examining the effects of aging in these strains would be appropriate because elderly populations appear to be specifically susceptible to air-pollutant–induced cardiac effects. Finally, the results of the present study suggest that genetic variability affects cardiac responses after acute exposures to air pollutants, such as PM and O_3_. Certain genetic polymorphisms have been associated with susceptibility to air-pollutant–induced HRV changes (Park et al. 2006; Schwartz et al. 2005b). The genetic determinants underlying the dramatic HRV differences between B6 and C3 mice in response to O_3_ exposure warrant further investigation.\

## Figures and Tables

**Table 1:** Homeostatic characteristics shown as mean values (± SE) for the circadian pattern results before each exposure protocol.

	Mouse strain		
Characteristic	B6	OuJ	HeJ
Body weight (g)			
Presurgical	29.2 ± 1.4	32.7 ± 0.6	29.7 ± 1.1
Pre-FAFA	29.1 ± 0.6	31.8 ± 0.8	30.4 ± 0.7
Pre-FACB	28.6 ± 0.7	31.9 ± 0.8	30.8 ± 0.9
Pre-O_3_CB	28.8 ± 0.6*	33.8 ± 1.1	31.8 ± 0.9
Body temperature (ºC)			
Pre-FAFA			
Mean	36.1 ± 0.1	36.3 ± 0.04	35.9 ± 0.1**
Maximum	37.5 ± 0.1	37.5 ± 0.1	37.1 ± 0.1**
Minimum	34.9 ± 0.1	35.4 ± 0.1**	34.6 ± 0.2
Pre-FACB			
Mean	36.1 ± 0.04	36.2 ± 0.1	35.8 ± 0.1**
Maximum	37.5 ± 0.1	37.5 ± 0.1	37.1 ± 0.1**
Minimum	34.9 ± 0.1	35.3 ± 0.1**	34.7 ± 0.2
Pre-O_3_CB			
Mean	36.2 ± 0.1	36.2 ± 0.1	35.8 ± 0.1**
Maximum	37.5 ± 0.1	37.5 ± 0.1	37.0 ± 0.2**
Minimum	34.9 ± 0.1	35.3 ± 0.1**	34.5 ± 0.2
HR (bpm)			
Pre-FAFA			
Mean	540 ± 10**	621 ± 5	623 ± 8
Maximum	670 ± 7**	742 ± 10	730 ± 3
Minimum	431 ± 11**	512 ± 11	503 ± 14
Pre-FACB			
Mean	541 ± 12*	631 ± 15	616 ± 16
Maximum	660 ± 12**	733 ± 14	728 ± 16
Minimum	439 ± 18*	535 ± 16	496 ± 25
Pre-O_3_CB			
Mean	573 ± 14*	648 ± 8	614 ± 17
Maximum	685 ± 12*	738 ± 8	722 ± 19
Minimum	466 ± 22*	561 ± 10	497 ± 20

* *p* < 0.01 vs. OuJ.

** *p* < 0.01 vs. the two other strains.

**Table 2:** HR, HRV, and VO_2_ responses (mean ± SE) for each exposure protocol at MB and MA.

		Protocol		
Characteristic	Time period	FAFA	FACB	O_3_CB
HR (bpm)				
B6	MB	557 ± 16	547 ± 20	559 ± 20
	MA	532 ± 24	493 ± 18*	489 ± 27*, #
OuJ	MB	598 ± 15	586 ± 15	587 ± 12
	MA	581 ± 28	589 ± 37	564 ± 17
HeJ	MB	540 ± 7	555 ± 16	558 ± 14
	MA	539 ± 13	535 ± 12	525 ± 19
SDNN (msec)				
B6	MB	12.8 ± 0.8**	13.4 ± 0.6**	13.4 ± 1.6**
	MA	13.0 ± 1.1**	13.7 ± 0.7**	14.8 ± 1.2**
OuJ	MB	5.1 ± 0.4	4.9 ± 0.3	5.2 ± 0.6
	MA	5.6 ± 0.5	5.1 ± 0.8	6.9 ± 0.9
HeJ	MB	7.9 ± 0.7	7.2 ± 0.5	7.0 ± 0.3
	MA	8.2 ± 0.8	6.8 ± 0.6	9.1 ± 0.7
RMSSD (msec)				
B6	MB	8.4 ± 1.4*	8.9 ± 1.3**	9.1 ± 1.9**
	MA	8.5 ± 1.4*	8.8 ± 1.6**	10.0 ± 1.3**
OuJ	MB	2.7 ± 0.3	2.6 ± 0.2	3.1 ± 0.5
	MA	3.0 ± 0.4	2.7 ± 0.5	4.9 ± 0.9
HeJ	MB	4.5 ± 0.9	3.7 ± 0.4	3.3 ± 0.2
	MA	4.9 ± 0.7	3.8 ± 0.4	4.7 ± 0.1
LF/HF				
B6	MB	1.2 ± 0.2	1.0 ± 0.1	1.3 ± 0.1
	MA	1.4 ± 0.2*	1.5 ± 0.5*	2.1 ± 0.5
OuJ	MB	2.1 ± 0.2	1.9 ± 0.3	3.1 ± 0.5**
	MA	2.7 ± 0.5	2.9 ± 0.8	1.5 ± 0.3#
HeJ	MB	1.2 ± 0.3	1.7 ± 0.3	1.6 ± 0.2
	MA	1.4 ± 0.2	1.6 ± 0.5	2.0 ± 0.3
VO_2_ (L/kg/hr)				
B6	MB	2.99 ± 0.27	3.11 ± 0.16	3.12 ± 0.28
	MA	2.52 ± 0.30	3.07 ± 0.22	2.88 ± 0.27
OuJ	MB	2.44 ± 0.23	2.84 ± 0.06	2.78 ± 0.21
	MA	2.96 ± 0.59	2.81 ± 0.14	2.97 ± 0.28
HeJ	MB	2.64 ± 0.22	3.03 ± 0.09	2.94 ± 0.13
	MA	2.76 ± 0.21	3.00 ± 0.18	2.79 ± 0.28
O_2_ pulsem (mL/kg/hr/bpm)				
B6	MB	5.28 ± 0.31	5.80 ± 0.21	5.59 ± 0.38
	MA	4.73 ± 0.37	6.14 ± 0.26##	5.80 ± 0.37
OuJ	MB	4.08 ± 0.37	4.85 ± 0.11	4.71 ± 0.29
	MA	4.97 ± 0.81	5.04 ± 0.12	5.50 ± 0.26
HeJ	MB	4.89 ± 0.42	5.51 ± 0.31	5.29 ± 0.29
	MA	5.09 ± 0.30	5.59 ± 0.25	5.32 ± 0.54

* *p* < 0.01 vs. OuJ.

** *p* < 0.01 vs. the two other strains.

# *p* < 0.01 MB versus MA.

## *p* < 0.01 vs. FAFA.
